# Occupational Disorders, Daily Workload, and Fitness Levels Among Fitness and Swimming Instructors

**DOI:** 10.3389/fpubh.2021.666019

**Published:** 2021-06-09

**Authors:** Giampiero Merati, Matteo Bonato, Luca Agnello, Dorothée Grevers, Hanns-Christian Gunga, Stefan Mendt, Martina Anna Maggioni

**Affiliations:** ^1^Department of Biotechnology and Life Sciences (DBSV), University of Insubria, Varese, Italy; ^2^IRCCS Fondazione Don Carlo Gnocchi, Milan, Italy; ^3^IRCCS Istituto Ortopedico Galeazzi, Milan, Italy; ^4^Department of Biomedical Sciences for Health, Università degli Studi di Milano, Milan, Italy; ^5^Istituto di Medicina dello Sport di Milano, Milan, Italy; ^6^Charité – Universitätsmedizin Berlin, Corporate Member of Freie Universität Berlin and Humboldt-Universität zu Berlin, Center for Space Medicine and Extreme Environments, Institute of Physiology, Berlin, Germany

**Keywords:** occupational disorder, cardiopulmonary assessment, rate of perceived exertion, musculoskeletal disorders, heart rate monitoring

## Abstract

Few data have been published on occupational disorders among sports instructors, especially regarding those who are expected to continuously practice while teaching. As the number of sports instructors increases, new specific information about their possible injuries, daily workload, and fitness levels is needed. The aim of this study was to assess occupational disorders, cardiorespiratory fitness, and daily workload of fitness (FI) and swimming instructors (SI). An online survey addressing occupational disorders was conducted among 435 instructors (256 FI and 179 SI). In one subgroup (57 FI and 42 SI), cardiorespiratory fitness levels were evaluated using maximal oxygen consumption ($\overset{∙}{\text{V}}$O_2max_) as an indicator. Daily workload was assessed by monitoring the heart rate and perception of exertion (using the Borg scale). Of the two groups, FI exhibited a higher 2-year prevalence of musculoskeletal injuries and SI experienced more upper respiratory tract infections. $\overset{∙}{\text{V}}$O_2max_ ranged from 47.0 to 51.9 ml·kg^−1^·min^−1^ and was similar for both FI and SI. Regarding the daily workload, female SI had significantly higher mean heart rate and mean heart rate to maximal heart rate ratio compared to female FI, but no significant differences between male FI and SI were found. No significant differences were observed between the perceived exertion of FI and SI. Preventive strategies for the reduction of occupational disorders in FI and SI are needed.

## Introduction

The fitness industry is booming: the last 10 years have shown a rapid increase in the number of fitness club members and employees, with almost 750,000 fitness employees in Europe alone (1). In particular, the fitness industry includes both fitness and/or swimming pool centers. In fitness centers, customers can find lots of equipment for cardio and weight training or group fitness classes. In swimming pool centers, customers can find different types of swimming classes as well as water aerobics courses. Fitness (FI) and swimming instructors (SI) are the two different types of trainers usually employed at these centers. FI generally lead, instruct, and motivate individuals or groups in exercise activities, including cardiovascular exercises, strength training, and stretching. Typically, FI work with individual clients to design, explain, and demonstrate various exercises and routines, or they teach group classes where they organize and lead fitness lessons lasting 30–90 min. In these classes, instructors may set the music and choreography to exercise sequences, while often also using specific exercise equipment (e.g., stationary bicycles, weights, etc.). SI generally help people learn how to swim, improve swimming skills, and exercise in water. Moreover, they are also specialized in teaching water aerobics classes during which they perform aerobic exercises along with the water-immersed participants. These classes focus on aerobic endurance, creating an enjoyable atmosphere with music.

Both FI and SI professions are physically demanding. Most of the time, FI and SI find themselves having to carry out the exercises themselves while teaching, and they may work nights, weekends, holidays, and may even have to travel to different gyms or to clients' homes to teach classes or to conduct personal training sessions. However, despite the numerous health-related advantages of physical activity and exercise, the risk of occupational disorders among FI and SI has been poorly addressed by academic research. To date, a high prevalence of musculoskeletal injuries in FI has been described (2–7): most of the injuries were to lower arms and lower back (12.9%), and they were associated with the number of times per week the instructors exercised. No clear relationship between musculoskeletal disorders and sex has been uncovered thus far (7).

In addition to overall stress on the musculoskeletal system, FI and SI actively have to make use of their voices during classes and rely on their voices in a similar way that vocal performers, classroom teachers, salespeople, and others in vocally demanding professions do (8). FI and SI have reported voice difficulties that appear to be the result of an interaction between both environmental and physiological stress placed on the voice given that speaking/shouting and vigorous exercise often have to occur simultaneously during classes (9–11). Indeed, for FI and SI the voice is an essential professional asset used not only to provide education and direction but also to motivate and encourage class participants to persevere (12). The vocal effectiveness of the instructor has a direct influence on the satisfaction of clients and keeps them motivated to return. Furthermore, the amount and type of verbal motivation required are often driven by the fitness genre (8). There are also several factors that can add additional vocal strain, because instructors often vocalize with music and other noise sources in the background and often teach in acoustically poor spaces (12). As such, several papers found that fitness instructors experienced more hoarseness and episodes of voice loss during and after instructing and had a significantly higher prevalence of laryngeal nodules (8, 9, 12). Finally, gender was shown to impact the occurrence of vocal disorders with females being most commonly affected (13).

Since studies on the occupational disorders experienced by FI and SI are limited, the primary purpose of this study was to investigate the 2-year prevalence of occupational disorders experienced by FI and SI employed in various fitness center companies through a self-reported questionnaire. In addition, we assessed the fitness levels, workloads, and perceived exertion during a typical workday of SI and FI in order to explore the possible factors associated with occupational disorders in these occupations. In particular, the possible effects of sex, instructor type, and years of work experience on the observed occupational disorders were considered.

## Methods

### Study Design

In order to assess the 2-year prevalence of occupational disorders experienced by FI and SI, a retrospective, cross-sectional, self-reporting study was conducted. Subsequently, to investigate physical fitness and daily workload during a typical workday of FI and SI, a prospective, cross-sectional, substudy was performed. The outcome of the study was the prevalence of occupational disorders among FI and SI. We calculated a sample size taking the expected proportion of cases from previous literature data on musculoskeletal (7) and vocal disorders (8–11, 13) among FI. The calculation led to a corresponding number of 270 and 354 individuals, respectively (which included a predicted 20% rejection rate), which was needed to estimate the prevalence of musculoskeletal and vocal disorders.

### Participant Screening

Participants were recruited from various fitness centers located in the North of Italy. These centers employed both FI (e.g., dance aerobics, step aerobics, spinning, pilates, yoga, low-back pain exercise classes, strength training, boxing/kickboxing) and SI (e.g., water aerobics, swimming courses, mother/baby swimming courses). The questionnaires were collected from 2008 to 2010 by activating the communication channels of the sports centers and getting University students of the Faculty of Exercise Sciences in Milan to directly contact their colleagues at the sports centers where they attended classes. The inclusion criteria were being an FI or SI instructor and teaching a minimum of one class a week. The exclusion criterion was being unable to fill out the questionnaire, of which the English translation can be found in the Supplementary Material. Potential participants' e-mail addresses were provided by the head of each center. The responders were contacted by email where they were fully informed about the study's procedures and the benefits and risks associated with participation. A consent form was sent to the participant *via* e-mail which then had to be signed and e-mailed back by the participant. At this point, the online survey was e-mailed to the participant who had agreed to participate and met the above inclusion criteria. Participants who agreed to also participate in the next part of the study (i.e., the physical fitness assessment) were contacted by phone to organize the laboratory testing and daily workload monitoring.

### The Online Survey

The online survey was created according to the guidelines provided by Artino et al. (14). Subjects were requested to complete the survey within a 2-week period. The data retrieved from the online questionnaires was subsequently entered in a protected database, from which the data was reorganized in tabular form for the purpose of descriptive statistics. The survey requested information regarding personal physical data as well as the frequency, duration, and time period (early morning, morning, afternoon, or evening) of class participation. For the purposes of this study, all self-reported occupational disorders related to their work during the last 2 years were asked by having the participant answer the following question: “Have you experienced any occupational disorder as a FI or SI during the last 2 years?” (7). In the case of a positive response, participants had to specify each injury and the type of injury (acute/overuse) in accordance with the definitions provided by a consensus statement regarding disorder registration (15). A “disorder” was defined as any condition causing pain and/or limiting activity. Only those participants who saw a physician for their disorders were asked to report a diagnosis. Participants who did not see a physician were asked to report the location of the disorder. The extent of the disorder was examined by contingency questions regarding the limitation(s) that the injury placed on activity. The survey took 20–25 min to fill out.

### Physical Fitness Assessment

Participants' physical fitness was assessed using maximal oxygen consumption assessment ($\overset{∙}{\text{V}}$O_2max_) in the laboratory of the University of Milan upon individual appointment.

The cardiopulmonary exercise testing was performed after the physical assessment session during work in order to avoid the possible carryover effect of fatigue on the subsequent working days. Oxygen consumption ($\overset{∙}{\text{V}}$O_2_), carbon dioxide production ($\overset{∙}{\text{V}}$CO_2_), and pulmonary ventilation ($\overset{∙}{\text{V}}$E) were measured using a metabolic device on a breath-by-breath basis (Quarkb^2^, Cosmed, Rome, Italy) during a graded ramp cycle ergometer test (Monark Ergomedic mod. 839E, Monark, Vansbro, Sweden). All tests were carried out in a well-ventilated laboratory under standardized constant ambient conditions (i.e., a temperature of 22 ± 2°C and humidity of <70%). The protocol consisted of 3 min at 50 W/min (warm-up and familiarization), followed by an increase of 20 W every min until exhaustion. Achievement of $\overset{∙}{\text{V}}$O_2max_ was considered as the attainment of at least two of the following criteria: (1) a plateau of $\overset{∙}{\text{V}}$O_2_ levels despite increasing speed; (2) a respiratory exchange ratio above 1.1; and (3) a heart rate (HR) of ±10 bpm of age-predicted maximal HR (i.e., 220−age) (16). HR was recorded during the entire test using an HR monitor (Polar RS800, Polar Electro, Kempele, Finland). Maximal HR at exhaustion was considered as HR_max_.

### Daily Workload Monitoring

Each participant was equipped with the HR monitor and instructed to wear it during their typical workday for 1 week. We then evaluated the day of greatest work commitment of the week for each individual, from which we retrieved the HR during the peak 3 h of the effective work hours. HR recordings were expressed as the percentage of the maximum value (% HR_max_) reached during the maximal oxygen consumption assessment. All the HRs obtained were then compared to the American College of Sports Medicine's recommendations (17) for the development of aerobic fitness, which define the relationship between work HR ranges and work intensity. Participants were asked to continue their normal daily working routine and to maintain their usual diets during the monitoring period.

### Rating of Perceived Exertion Assessment

The Borg CR100 scale (18) was selected to rate the perceived exertion of a typical lesson. A verbal-anchored scale was provided to the participants who were instructed to use it 30 min after the end of their workday. Each participant was preliminary familiarized with the Borg CR100 scale, including anchoring procedures.

### Statistical Analysis

Respondents with missing data were excluded from the analysis. Descriptive statistics (mean ± standard deviation) for the outcome measures were calculated. The normality of the distribution was checked using the Kolmogorov–Smirnov test. Since all anthropometric variables were normally distributed, differences between male and female FI and SI were checked using an unpaired Student's *t*-test. A Chi-square test was used to compare the questionnaire's variables of educational level, professional information, and job characteristics between FI and SI groups. Differences between the perceived exertion after the maximally fatiguing workday and the perceived exertion 30 min after the end of the lessons of FI and SI were studied using the Student's paired *t*-test. Intra- and intergroup differences (gender × instructor type) for $\overset{∙}{\text{V}}$O_2max_, HR_mean_, and HR_max_ between daily workload for FI and SI were checked using two-way analysis of variance with Bonferroni's multiple-comparison test. The level of statistical significance was set at *p* < 0.05. Statistical analysis was performed using the software STATISTICA (version 7.1, StatSoft, Tulsa, OK, USA).

## Results

### Participant Screening Results

**Figure 1:**
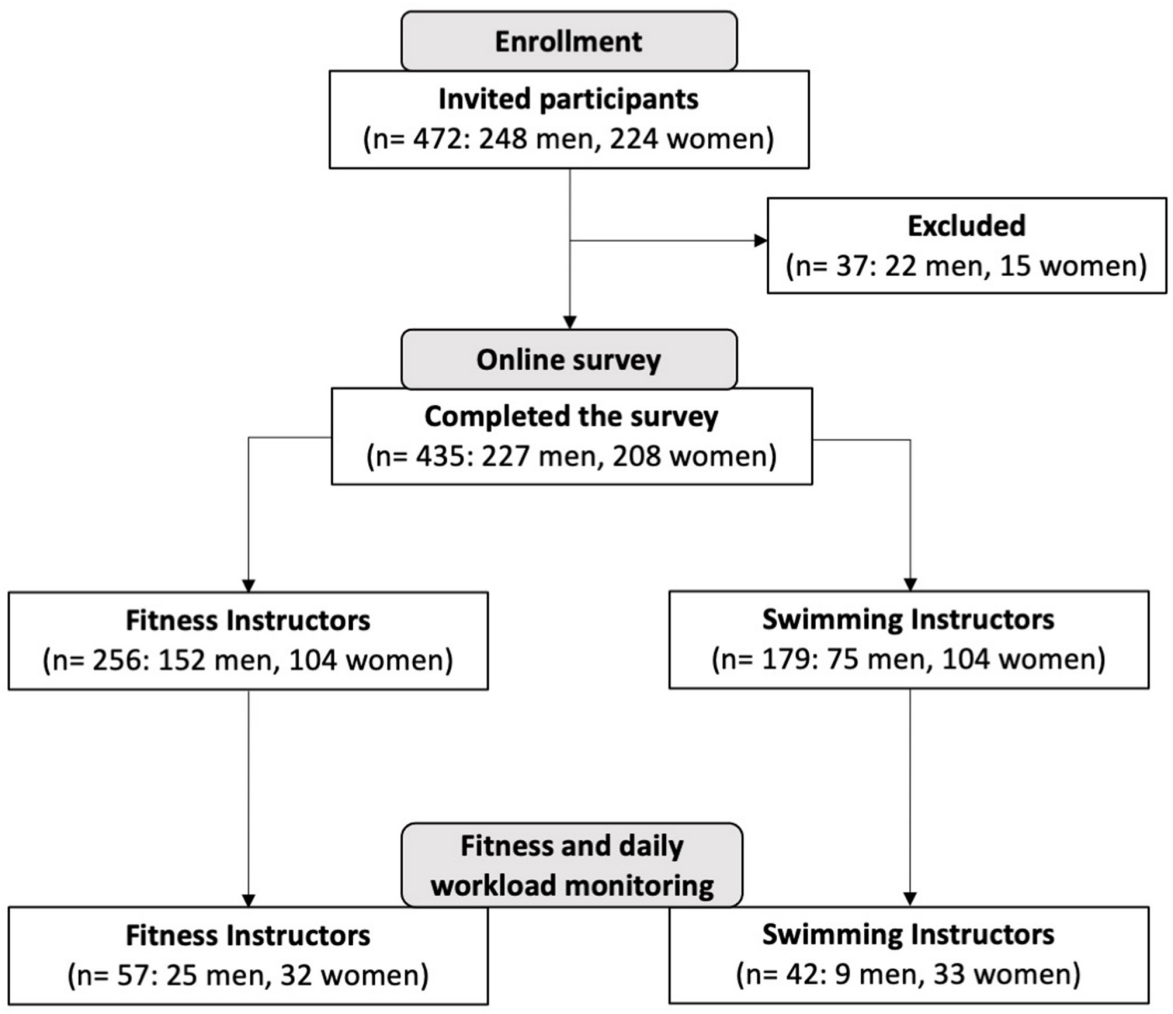
Study flow diagram.

The study population included 472 participants of which 435 instructors completed the online survey, (response rate: 92.2%). Ninety-nine subjects (57 FI and 42 SI) agreed to also participate in the next phase of the study during which their fitness level, workload monitoring, and perceived exertion of a typical workday were measured. The design of the study is shown in Figure 1.

### Online Survey Results

Table 1 shows the demographics of the participants. The participants in each group were similar in age, height, body mass, and body mass index (BMI). Only a small percentage of the participants had been active in their profession >10 years. The number of weekly work hours was <10 h for more than half of all FI and between 10 and 30 h per week for all SI. The presence of preexisting pathologies was not investigated in this study. Most of the participants (~70%) stated that they partake in both competitive and/or noncompetitive sports outside of their profession for an average of 5.4 ± 4.2 h per week. In Italy, SI and FI are obligated to obtain annual certificates of good health from a sports medicine physician in order to practice their profession. The annual visit includes an accurate medical history, an ECG at rest (and during exercise in case of agonistic activity), a spirometric evaluation, and a clinical physical examination.

**Table 1:** Results of whole sample questionnaires regarding demographics and job characteristics.

	**FI**	**SI**
**Demographic and general characteristics**		
All (*n*)	256	179
Men (*n*, %)	152, 59	75, 41
Women (*n*, %)	104, 41	104, 59
Age (years)	28 ± 7	30 ± 8
Height (m)	1.73 ± 0.08	1.70 ± 0.09
Body mass (kg)	67.3 ± 12.1	64.5 ± 11.5
BMI (kg·m^−2^)	22.2 ± 2.4	22.1 ± 2.7
**Job type**		
FI/SI as main occupation (%)	41	46
FI/SI as secondary occupation (%)	59	54
**Career duration**		
<5 years (%)	52	41^*^
5–10 years (%)	33	31
>10 years (%)	15	28^**^
**Weekly working hours**		
<10 h (%)	51	31^**^
10–30 h (%)	37	54^**^
>30 h (%)	12	15

*FI, fitness instructors; SI, swimming instructors.*

* *p < 0.05 between groups;*

** *p < 0.01 between groups*.

**Figure 2:**
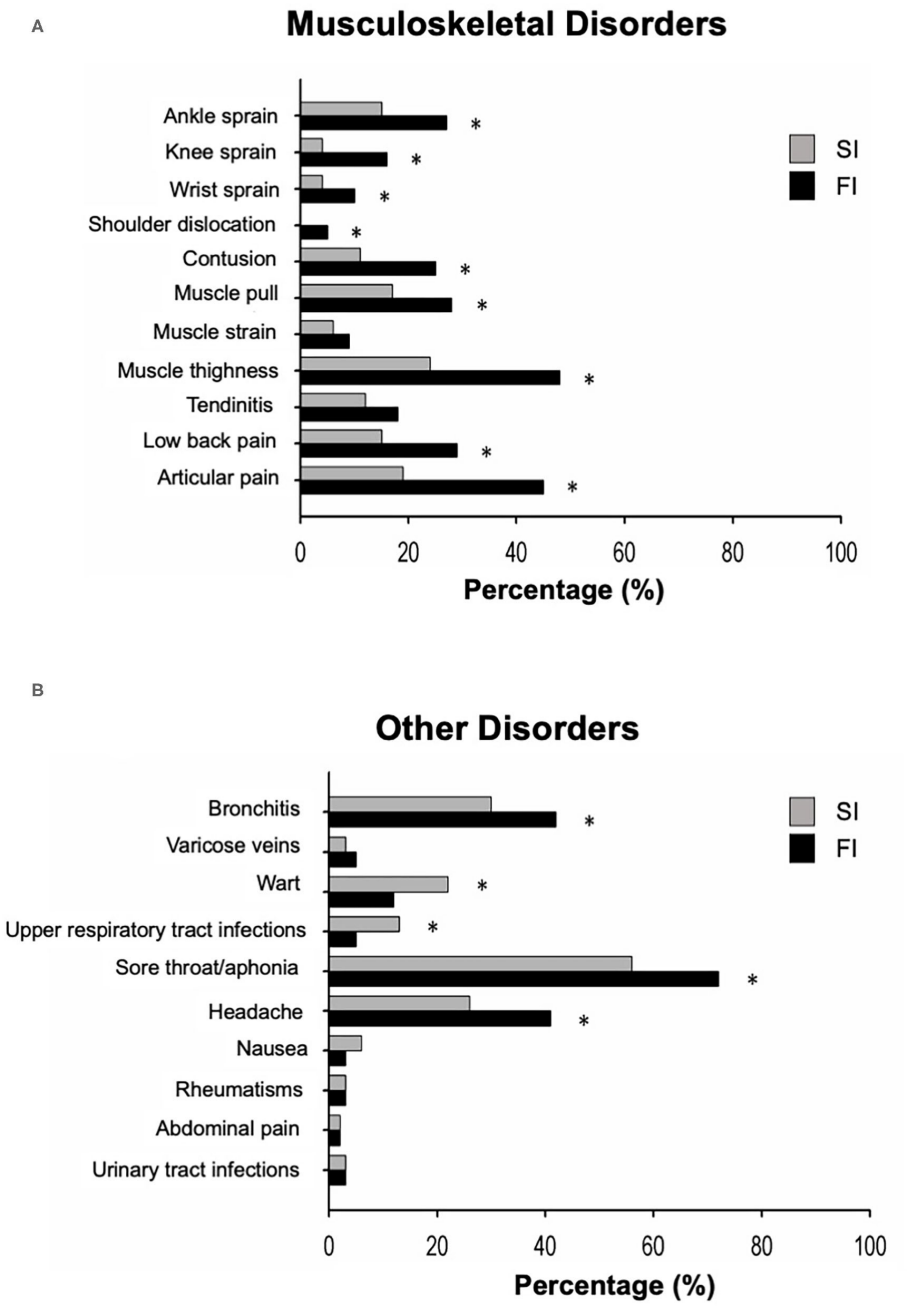
The figure represents the proportion of individuals in the swimming and fitness instructor groups (SI, swimming instructors, *n* = 179; FI, fitness instructors, *n* = 256), who experienced specific musculoskeletal injury **(A)** or other disorders **(B)** in the last 2 years. *p < 0.05 between groups.

Overall, a total of 621 musculoskeletal disorders and 521 other types of disorders were reported in the study of 157 FI (61% of 256 FI who completed the survey) and 155 SI (86% of 179 SI who completed the survey), that experienced two or more injuries during the last 2 years. Figure 2 illustrates the 2-year prevalence of occupational disorders that occurred in the FI and SI careers, divided into musculoskeletal and other disorders. The percentages of ankle, knee, and wrist sprains, shoulder dislocations, contusions, muscle pulls and contractures, lower back pain, and articular pain were significantly higher in the FI group vs. the SI group (*p* = 0.032). Non-musculoskeletal diseases such as bronchitis, sore throat/aphonia, and headache were significantly more common in the FI group (*p* = 0.014), whereas warts and upper respiratory tract infections were more frequent in the SI group (*p* = 0.025).

### Physical Fitness Results and Daily Workload Monitoring Results

FI and SI groups did not differ significantly in $\overset{∙}{\text{V}}$O_2max_. The FI and SI $\overset{∙}{\text{V}}$O_2max_, HR at rest, and HR_max_ classified by sex and instructor type are shown in Table 2. FI and SI groups did not differ significantly in $\overset{∙}{\text{V}}$O_2max_, HR at rest, and HR_max_, and no interaction between sex and instructor type was observed between groups. Additionally, the HR_mean_ during 3 h of a typical workday and the ratio between HR_mean_ and HR_max_ are shown in Table 2. While there was no significant main effect found between male SI and FI, the female SI displayed significantly higher HR_mean_ and HR_mean_/HR_max_ than the female FI did (*p* = 0.018, *p* = 0.022, respectively).

**Table 2:** Physiological variables during maximal oxygen consumption ($\overset{∙}{\text{V}}$O_2max_) assessment and heart rate (HR) data during daily workload monitoring of fitness instructors (FI) and swimming instructors (SI).

**Parameter**	**Males**		**Females**	
	**FI (*n* = 25)**	**SI (*n* = 9)**	**FI (*n* = 32)**	**SI (*n* = 33)**
**Exercise test**				
HR_rest_ (beats·min^−1^)	66 ± 14	62 ± 14	66 ± 13	65 ± 10
HR_max_ (beats·min^−1^)	186 ± 5	186 ± 4	187 ± 4	187 ± 5
$\overset{∙}{\text{V}}$O_2max_ (mL·kg^−1^·min^−1^)	51.9 ± 3.7	50.9 ± 3.8	48.9 ± 3.6	47.0 ± 4.0
**Daily HR recording**				
HR_mean_ (beats·min^−1^)	127 ± 28	144 ± 6	126 ± 21	139 ± 19^*^
HR_mean_/HR_max_	0.69 ± 0.14	0.78 ± 0.40	0.68 ± 0.11	0.75 ± 0.10^*^

* *p < 0.05 between groups*.

### Rating of Perceived Exertion Results

**Figure 3:**
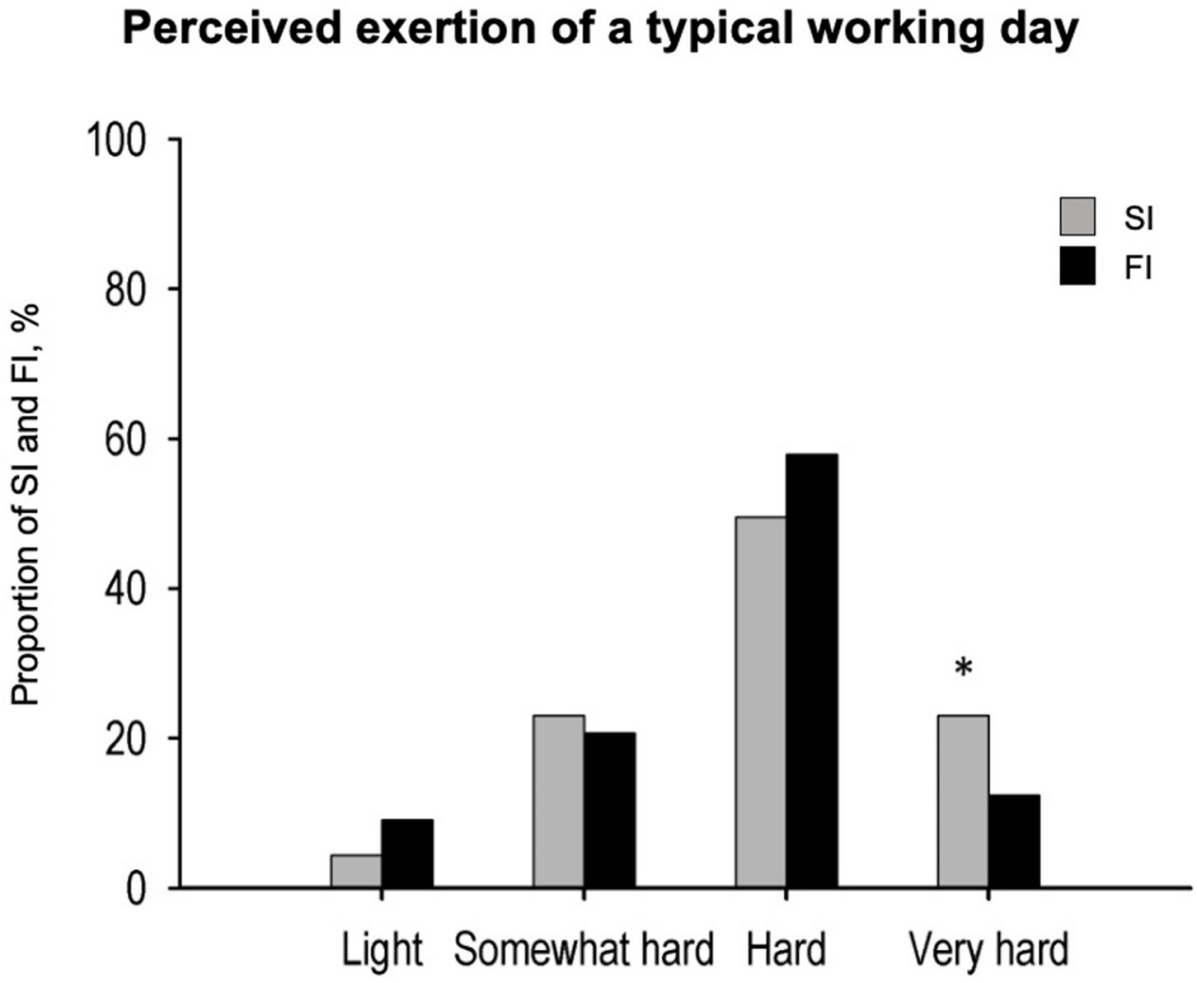
Perceived exertion during a typical lesson (SI, swimming instructors, *n* = 42; FI, fitness instructors, *n* = 57). *p < 0.05 between groups.

The perceived level of exertion after a typical workday was 72.3 ± 16.2 AU (i.e., arbitrary units in the CR100 scale, a point scale up to 100 with 100 being the maximum possible level of exertion) (18) in FI and 72.0 ± 18.0 AU in SI, with no significant differences between the groups. Figure 3 shows the perceived physical exertion of a typical lesson conducted by FI and SI. About 50% of the SI group and 60% of the FI group reported feeling that their typical lesson was physically “hard,” with no significant differences between groups. A significantly higher percentage of SI participants described the physical exertion of their lesson as “very hard” (*p* = 0.042 between groups, χ^2^ test for percentages).

## Discussion

To the best of our knowledge, the present study is one of the first that investigated the prevalence of occupational disorders among FI and SI. In particular, we observed that FI had a higher 2-year prevalence of musculoskeletal occupational disorders, whereas SI experienced more acute and chronic voice disorders. Moreover, since FI and SI have to cope with physical exertion and psychological stress, we have provided objective data on their physical fitness level and workload during a typical workday.

Regarding musculoskeletal disorders, we observed that muscle tightness (i.e., a shortening of a muscle), ankle, knee, and wrist sprains, shoulder dislocations, contusions, low-back pain, and articular pain were very common among FI. Our results are in line with previous findings on FI occupational health (3–5, 7, 19). Hickey and Hager (19) showed that the most common chronic injuries in aerobic dance instructors were tendinitis, repetitive strain injury, patello-femoral diseases, and medial tibial syndromes, followed by ankle sprain and low-back pain, as suggested by Rothenberger et al. (3). Also, du Toit et al. (4) and Bratland-Sanda et al. (7) reported that the lower-limb injuries were very common, with the ankle (32.8%) and the knee (20%) being the most common sites of injury. Generally, these types of injuries are classified as overuse injuries, resulting from repetitive force applied to a tissue, joint, or ligament. Bratland-Sanda (7) stated that the greater risk of lower-limb musculoskeletal disorders in FI is related to the monotonous exercise modality, which is a primary risk factor for overuse injuries. In addition, Sohl and Bowling (17) reported high-intensity training classes, unsuitable floors, shoe type, high number of workouts per day, difficult choreography, and insufficient warm-up as the factors that may contribute to more occupational disorders of the lower limbs. Finally, Scharff-Olson (20) indicated that the number of weekly classes was an additional variable associated with musculoskeletal disorders. In fact, four aerobic dance sessions per week increased the injury incidence from 43% to 66% compared to subjects who exercised three times per week or less (20). On the contrary, we found that SI had a lower prevalence of musculoskeletal occupational disorders. This was not unexpected seeing as SI work is largely done standing (e.g., classic swim classes) or is anti-gravitational (e.g., during water immersed aerobic classes).

With regard to the other disorders, the present investigation found that both FI and SI are at a higher risk of developing both acute and chronic voice difficulties associated with the development of sore throat, aphonia, and bronchitis. These results corroborate previous research that found that 58 and 12% of group fitness instructors experience hoarseness and voice loss immediately following classes (20). It seems reasonable to associate these disorders with the typical demands of the job that require loud verbal instructions while performing exercises, thereby making the control of breathing and airflow movement more stressful. Indeed, it has been demonstrated that the interaction between both environmental and physiological stress leads FI and SI to assume a hyperfunctional behavior that could also be worsened by postural misalignment, breathing patterns, and work environment and therefore lead to the adoption of compensatory vocal behaviors (21). This has been observed especially in young and inexperienced instructors who risk developing voice overuse and laryngeal diseases in the long run (22). Another incidental factor may be the poor air quality (e.g., dryness, dust) in the workplace that may cause allergic reactions or sinus infections (23). Finally, the use of chlorine-based products to sanitize swimming water in daily life may affect the respiratory health of SI (24). Moreover, we observed that SI are at a higher risk to develop headache and warts compared to FI. Regarding headaches, we hypothesize that the warm temperatures and humidity typical of swimming pool environments may play a role, especially in individuals prone to migraine attacks (25). Regarding warts, it is well known that swimming pools may be a more favorable environment for these types of infections (26).

Regarding the fitness level assessment, we are now able to provide evidence of the physical fitness and daily workload of FI and SI. In particular, we found that FI and SI showed the same $\overset{∙}{\text{V}}$O_2max_ during a graded maximal test and HR during a typical workday. Therefore, the aerobic fitness level was comparable between FI and SI subjects, suggesting that both groups are probably exposed to a similar workload, and thus training, during a workday. Our results are similar to those found in the study of Wanke et al. (27), who assessed the work-related cardiovascular loads in professional dance teachers. They found that, depending on the dance style (e.g., jazz, modern dance, ballet, etc.), the average HR load during the lessons ranged between 56.7 and 63.6% of the individual HR_max_. Interestingly, we found a significantly higher HR_mean_ in women during a typical workday in SI with respect to FI. We could therefore speculate that female SI are more often involved in aqua gym classes or similar training sessions, which require active physical participation from the instructor, whereas male SI are more likely to be devoted to swimming instruction or training, which does not include active physical involvement.

This study had some limitations. Firstly, the questionnaire we used was custom-made and has not yet been validated nor checked for internal consistency. After its design, the questionnaire was only submitted to a small group of fitness experts, who evaluated whether the questions effectively captured the topic under investigation. The data obtained with this questionnaire should therefore be considered as pilot data.

Secondly, due to the paucity of research in this area, the first part of this study was designed as a cross-sectional and exploratory study. Although this design is less expensive and can be performed within a shorter period of time, some confounding factors such as history of injuries and work habits prior to data collections cannot be controlled. Therefore, antecedent–consequent relationships as well as occupational disorders and relative risk cannot be established through this design. Thirdly, it was not possible to perform analysis of differences between respondents and nonrespondents. A possible selection bias is that the prevalence of injuries and musculoskeletal pain might have been higher among the respondents compared to the nonrespondents, thus affecting the results and the external validity of the study. Finally, the self-reporting of injuries and musculoskeletal pain is also a limitation, since this method makes it impossible to verify the injury location and type by a third party. However, the assessment of physical fitness of FI and SI as well as daily workload and their perceived exertion are valuable information to focus on and when designing future studies. We therefore suggest that future research considers these factors to conduct more meaningful longitudinal studies on this topic.

## Conclusion

In conclusion, a high 2-year prevalence of instruction-related musculoskeletal disorders and vocal pathologies was observed in FI and SI, respectively. The role of the work environment should be considered as an occupational hazard. Guidelines on the maximum weekly instruction load are therefore recommended for SI and FI professionals.

## Data Availability Statement

The data supporting the conclusions of this article will be made available by the authors, upon request, without undue reservation.

## Ethics Statement

The studies were reviewed and approved by Scientific and Technical Committee of the ISPESL (Istituto Superiore Prevenzione e Sicurezza sul Lavoro, Italian Ministry of Health, n° B19/DOC/03). The participants provided their written informed consent to participate in this study.

## Author Contributions

MAM designed the study (with Prof. Arsenio Veicsteinas) and performed the data collection. MB, LA, GM, and SM analyzed the data, provided the figures, and together with DG wrote up the manuscript. MAM, H-CG, and GM did the critical revision of the manuscript. All authors contributed to the article and approved the submitted version.

## Conflict of Interest

The authors declare that the research was conducted in the absence of any commercial or financial relationships that could be construed as a potential conflict of interest.
