# Supplementary material for: Occupational Disorders, Daily Workload, and Fitness Levels Among Fitness and Swimming Instructors
Source: Front Public Health. 2021 Jun 9;9:666019. doi: 10.3389/fpubh.2021.666019 (PMC8217866; doi:10.3389/fpubh.2021.666019)
Supplement: Supplementary file 1 [file Data_Sheet_1.PDF]

Research project on the professional commitment of sports operators
Institute of Physical Exercise, Health and Sports Activity (IEFSAS), University of Milan
ANONYMOUS QUESTIONNAIRE (even more answers are allowed)

Section A - Personal data and anamnesis

1. Code (leave blank) ____________

2.a) Year of birth ___________________________________

b) Place of birth (= Country - if Italy only Province): ________________________________

3.a) Ethnicity
- Asian ☐
- African American ☐
- White Caucasian ☐
- Hispanic ☐

b) Sex M ☐ F ☐

4.a) Education:

- Compulsory education ☐
- High School ☐
- University Bachelor's Degree ☐
- University Master's Degree ☐
- Other (eg. Qualification obtained abroad) ☐

b) If university degree or diploma, which one:

- ISEF (i.e. Superior Institute for Exercise Sciences) Diploma ☐
- Rome Sports School Diploma ☐
- Exercise Sciences Bachelor's Degree ☐
- Master's Degree in Exercise Science ☐
- Diploma / Degree in physical education obtained abroad. ☐
- Other ☐

c) Year of graduation: ____________________________

5.a) List one at a time the PROFESSIONAL TRAINING COURSES attended in the last 3 years (specify the organization organizing the course)
Ex: Lifeguard Assistant Course (F.I.N.), Spinning Instructor Course (BCUBE), etc.

____
____
____
____

b) List one at a time the REFRESHER COURSES attended in the last 3 years (specify the organizing body of the course) Eg. Masterclass *Acquafitness* (EAA), Refresher Course for Step & Aerobics Instructors (FIA), etc.

____
____
____

6. Current main profession:

- Student ☐
- Worker ☐
- Employee ☐
- Professional ☐
- Fitness operator ☐
- Swimming pool operator ☐
- School teacher - ISEF ☐
- Ski instructor ☐
- Coach ☐
- Other ☐ describe ______________________________

7. Height, cm ______________

8. Weight, kg ______________

9. Do you smoke?

Yes ☐ - currently specify how many cigarettes: less than 5 per day ☐
from 5 to 10 per day ☐
more than 10 per day ☐

No ☐

Former smoker ☐ - specify by how long:

____

10. In your family there are cases of:

	Parents	Grandparents	Siblings
High blood pressure	☐	☐	☐
Diabetes Mellitus	☐	☐	☐
Cardiovascular disease (heart attack, stroke)	☐	☐	☐
Hypercholesterolemia	☐	☐	☐
Obesity	☐	☐	☐
Tumors	☐	☐	☐
Diseases of the blood	☐	☐	☐

11. Do you regularly take medications?

• Yes ☐ - specify which ones: ____
____
____

• No ☐

Section B - Work activity

12. In which year did you start your activity as a sports instructor? _______________

13. For the activities listed below that you regularly practice as an instructor, specify the number of hours you spend during the week:

Room instructor (body building, personal trainer, etc)	N hours / week ______________
Spinning Instructor	N hours / week______________
Aerobics Instructor (step, cardio kick, etc)	N hours / week ______________
Dance instructor (hip-hop, Caribbean, etc)	N hours / week ______________
Toning courses (gag, gab, tone up, pump, etc.)	N hours / week______________
Aquafitness courses instructor	N hours / week ______________
Lifeguard assistant	N hours / week______________
Children's swimming school instructor	N hours / week ______________
Adult swimming lessons instructor	N hours / week ______________
Aquatic courses instructor	N hours / week ______________
Parent-child course instructor	N hours / week______________
Pregnant courses instructor	N hours / week ______________
Disabled course instructor	N hours / week ______________
Instructor of functional re-education courses in water	N hours / week ______________
Coach (pre-competitive / competitive)	N hours / week ______________
Synchronized swimming instructor / coach	N hours / week ______________
Other (describe)	N hours / week ______________

____
____

14.<u>If</u> you are a teacher of Aquafitness, Spinning, land courses in general, for example aerobics, toning, dance etc. (if otherwise go to question 15):

a) How many consecutive hours are you on average engaged in activities such as Aquafitness, Spinning, land courses, etc.? (please specify the number precisely)

Number of hours: ______________________

b) The duration of the proposed lessons is:

☐ 30 min.
☐ 45 min.
☐ from 50 to 60 min.

c) There are breaks between one lesson and the next:

☐ Yes
☐ No

d) On a scale that goes from the minimum of physical-mental commitment and reaches the maximum, do you indicate how you physically perceive the conduct of a "typical" lesson of your main activity?
Physical-mental commitment
Very light *Light* Enough light *A little demanding* Demanding *Heavy* Very heavy
☐ ☐ ☐ ☐ ☐ ☐ ☐ ☐ ☐ ☐ ☐ ☐ ☐

15. In addition to your work as an instructor, do you regularly engage in any other physical activity?
☐ Yes - List the type and commitment (in weekly hours) of physical activities one at a time:
Activity 1: ________________________________ Number of hours/week______________
Activity 2: ________________________________ Number of hours/week______________
Activity 3: ________________________________ Number of hours/week______________

☐ No

16. Why did you choose to carry out this particular job?

Because it is the activity for which I completed my studies ☐
To stay fit ☐
To improve the physical appearance ☐
To stay in touch with others ☐
Because it's a job like any other ☐
Other (describe) ☐
____
____

17. Do you feel satisfied and satisfied with your activity as an instructor?

Completely ☐
In part ☐
Not much ☐
Not at all ☐

Section C - Consequences on health

18. With respect to your activity as an instructor, how do you rate your pace of work?

Psychologically stressful ☐
Psychologically relaxing ☐
Physically heavy ☐
Healthy and balanced ☐
Other (describe) ☐

____

____

19. How does the work environment affect your state of health (on your psycho-physical well-being)?

Positively ☐ because: ____
Negatively ☐ because: ____
It doesn't affect ☐

20.a) Have you complained of arthro-osteo-articular disorders during the last 2 years of activity as an instructor? (Please report on the side the number of experienced disorders)

	**No. 1-3**	**3-6**	**more than 6**
Ankle Sprains	☐	☐	☐
Knee sprains	☐	☐	☐
Wrist sprains	☐	☐	☐
Shoulder dislocations	☐	☐	☐
Elbow dislocations	☐	☐	☐
Bruises of various types	☐	☐	☐
Muscle strains	☐	☐	☐
Muscle damages	☐	☐	☐
Contractures	☐	☐	☐
Fractures	☐	☐	☐
Tendintis	☐	☐	☐
Back (Sciatic nerve) pain	☐	☐	☐
Joint pain	☐	☐	☐

b) Have the complaints you mentioned in question 20 recurred in the last 6 months?

☐ Yes - list which ones repeated: ______________________________
____

☐ No

21. a) Which of these disorders do you think are related to your activity as an instructor? (Please report on the side the number of times you have experienced the indicated disorder during the last 2 years of your career)

	**No. 1-3**	**3-6**	**more than 6**
Bronchitis, flu	☐	☐	☐
Circulation problems (varices of the lower limbs)	☐	☐	☐
Dermatological problems (warts, fungi,)	☐	☐	☐
Respiratory problems (laryngitis, tracheitis, asthma)	☐	☐	☐
Sore throat, hoarse voice, aphonia	☐	☐	☐
Headache, migraine, headache	☐	☐	☐
Nausea	☐	☐	☐
Rheumatism	☐	☐	☐
Kidney stones	☐	☐	☐
Abdominal colic	☐	☐	☐
Urinary tract infections	☐	☐	☐

b) Have the complaints you indicated in question 21 recurred in the last 6 months?

☐ Yes - list which ones repeated: ______________________________
____

☐ No

22. Have you ever experienced muscle weakness or fatigue in the past 6 months?

☐ Yes - how often? ☐ 1 to 3 times
☐ more than 3 times
☐ more than 6 times

☐ No

23. Did you develop allergies during your training as an instructor?

☐ Yes - Describe which ones: ______________________________

____

☐ No

24. Did you develop anemia during your training as an instructor?

☐ Yes
☐ No

***24. bis* (only for women)** Have you been found by your doctor, or have you accused of gynecological dysfunctions during your working career, dysfunctions that you believe are related to the work performed?

☐ None
☐ Amenorrhea (absence of menstruation)
☐ Dysmenorrhea (heavy and painful menstruation)
☐ Temporal irregularities of the cycle
☐ Spontaneous abortion
☐ Sterility and infertility

25. Was it essential to undergo clinical examinations, specific examinations, hospital admissions for any traumas and disorders accused during your work?

No exam	☐
Blood tests	☐
X-rays, resonances, CT scan	☐
Visit to a specialist (orthopedist, otolaryngologist, sports doctor, etc.)	☐
Hospital admissions	☐ - N: ___
Pharmacological treatments	☐
Other (describe)	☐

____

26. On a scale that starts from complete relaxation (= 0 - zero) and goes to the maximum of tiredness (= 100 - one hundred), where 50 indicates "normality", indicate with an X what is your level of fatigue IN THE EVENING THE DAY OF MAXIMUM WORKING ACTIVITY.

Evening of the most intense working day

maximum relaxation — no effect — maximum fatigue

|______________________________|______________________________|
|____|_____|______|______|_____|_____|______|_____|______|_____|
0 10% 20% 30% 40% 50% 60% 70% 80% 90% 100%

27. On a scale that starts from complete relaxation (= 0 - zero) and goes to the maximum of tiredness (= 100 - one hundred), where 50 indicates "normality", indicate with an X what your level of fatigue is IN THE MORNING ON THE DAY AFTER that of maximum working activity.

Morning after more intense working day

maximum relaxation — no effect — maximum fatigue

|______________________________|______________________________|
|____|_____|______|______|_____|_____|______|_____|______|_____|
0 10% 20% 30% 40% 50% 60% 70% 80% 90% 100%

28. How many liters of water do you drink on average per day (approximately)?

☐ 1
☐ 2
☐ 3
☐ 4
☐ 5
☐ 6

29. Do you use food supplements, multivitamin supplements, saline supplements, to balance the losses related to your teaching activity?

☐ Yes - Protein ☐
- Multivitamins ☐
- Mineral salts and electrolytes ☐
- Others ☐

☐ No

30. Add any additional information that you believe may be useful to better understand and define your work commitment in the field of fitness.

____

____

____

____

____

____

The processing of personal data and those deriving from the study will be kept anonymously and for research purposes only (pursuant to article 10 of law no. 675 of 31/12/1996 on the protection of personal data).

We warmly thank you for your cooperation.

Prof. Dr. ArsenioVeicsteinas
(Principal Investigator)
